# Implementation of Guideline-Directed medical therapy and factors in heart failure with reduced ejection fraction

**DOI:** 10.1186/s12872-025-05187-5

**Published:** 2025-10-09

**Authors:** Firaol Wassie Bekele¹, Wubshet Abraham Alemu, Koricho Simie Tolla, Abdulkadir Urgessa Jada, Mengesha Akale Tekle, Muhammedawel Kaso Adem, Hassen Ahmed Yesuf

**Affiliations:** 1 Department of Internal Medicine, College of Health Sciences, Arsi University, Asella, Ethiopia; 2 Department of Public Health, College of Health Sciences, Arsi University, Asella, Ethiopia; 3 Department of Biomedical Science, School of Medicine, College of Medicine and Health Sciences, Woldia University, Woldia, Ethiopia

**Keywords:** ARNI, Ethiopia, Guideline-directed medical therapy, Heart failure with reduced ejection fraction, Medication adherence, SGLT2 inhibitors

## Abstract

**Background:**

To evaluate the implementation of guideline-directed medical therapy and identify associated clinical factors among patients with heart failure with reduced ejection fraction (HFrEF) receiving follow-up at Asella Referral and Teaching Hospital, Ethiopia.

**Methods:**

We conducted a hospital-based cross-sectional study involving 156 patients with HFrEF attending follow-up at Asella Referral and Teaching Hospital from October to December 2023. Eligible participants were adults with LVEF ≤ 40%, ≥ 6 monthly clinic visits, and a recent echocardiogram within two years. Data were collected through structured interviews and chart review and analyzed using SPSS version 26.

**Results:**

Prescription rates for renin-angiotensin system inhibitors (RASI), beta-blockers, and mineralocorticoid receptor antagonists (MRA) were 98.1%, 96.8%, and 70.5%, respectively. However, only 30.8% achieved target doses of RASI, 1.3% for beta-blockers, and 67.3% for MRA. Only 1.3% were on all three therapies at guideline-recommended target doses. No patient received angiotensin receptor–neprilysin inhibitors (ARNI) or sodium-glucose co-transporter 2 inhibitors (SGLT2i), primarily due to cost and availability. In multivariable analysis, baseline bradycardia (adjusted odds ratio [AOR] 13.7; 95% CI 1.8–100.3; *p* = 0.010) and diabetes mellitus (AOR 4.5; 95% CI 1.13–17.9; *p* = 0.033) were significantly associated with achieving RASI target doses. Absence of prior heart failure hospitalization was associated with a lower likelihood of achieving the MRA target dose (AOR 0.4; 95% CI 0.18–0.9; *p* = 0.029).

**Conclusion:**

The implementation of guideline-directed medical therapy in HFrEF patients was suboptimal, with only 1.3% achieving target doses of all three available therapies. No patients received ARNI or SGLT2 inhibitors due to availability and cost barriers. Key clinical factors such as baseline heart rate, diabetes, and hospitalization history influenced medication optimization. GDMT optimization in HFrEF remains inadequate in Ethiopia. Policy interventions (e.g., formulary expansion) and structured titration protocols are urgently needed.

**Supplementary Information:**

The online version contains supplementary material available at 10.1186/s12872-025-05187-5.

**Authors**:

ORCID: https://orcid.org/0009-0005-0134-8935.

## Background

Heart failure (HF) based on the left ventricular systolic function has been classified into three subtypes, namely HF with reduced ejection fraction, HF with preserved ejection fraction, and HF with mid-range ejection fraction [1–3]. Heart Failure with Reduced Ejection Fraction (HFrEF) is a clinical syndrome characterized by typical symptoms and signs of heart failure accompanied by a left ventricular ejection fraction (LVEF) of 40% or less, indicating impaired systolic function of the heart [4]. Heart failure with reduced ejection fraction (HFrEF) remains a leading cause of morbidity and mortality worldwide, with disproportionately high impacts in low- and middle-income countries [5].

Guideline-directed medical therapy (GDMT) for the management of heart failure with reduced ejection fraction (HFrEF) is recommended by many societies and organizations [6]. It consists of five medications in four tablets, a combination frequently referred to as the ‘four foundational“ pillars’/“quadruple therapy,” which includes an angiotensin receptor neprilysin inhibitor (ARNI)—the combination of a neprilysin inhibitor and an angiotensin receptor blocker (ARB) in the form of sacubitril/valsartan—a beta-blocker (BB), a mineralocorticoid receptor antagonist (MRA), and sodium-glucose co-transporter 2 inhibitors (SGLT2i) [7]. The 2022 American Heart Association/American College of Cardiology (ACC)/Heart Failure Society of America guideline [3], the 2021 ESC Guidelines for the Diagnosis and Treatment of Acute and Chronic Heart Failure [2], and the 2017 ACC Expert Consensus Decision Pathway for Optimization of Heart Failure Treatment [8] recommend the use of this regimen to treat HFrEF.

A network meta-analysis of 57 randomized trials found that HFrEF patients receiving guideline-directed medical therapy (GDMT) with beta-blockers, renin-angiotensin system blockers with neprilysin inhibitors, and mineralocorticoid receptor antagonists (MRA) experience a significant reduction in mortality, with a 63% decrease compared to placebo [9]. However, different studies reported that many heart failure patients either do not receive GDMT or the recommended doses due to patient factors (comorbidities, age, frailty, poor adherence), treatment issues (intolerance, side effects), and healthcare system barriers (accessibility and availability) [10, 11]. This study evaluates GDMT implementation in Ethiopia, where access to newer therapies (e.g., ARNI/SGLT2i) is limited. By identifying the gaps in GDMT utilization, it provides valuable insights into the factors affecting treatment adherence and optimization in a resource-limited setting. The findings could guide local healthcare practices, improve patient outcomes, and inform policy decisions to enhance the delivery of evidence-based care for heart failure patients in Ethiopia and similar low-resource environments. Additionally, it contributes to the broader understanding of barriers to GDMT adoption in sub-Saharan Africa, where heart failure management is often suboptimal. Due to the unavailability of angiotensin receptor-neprilysin inhibitors (ARNIs) and sodium-glucose co-transporter 2 inhibitors (SGLT2is) in Ethiopia, this study focused on the optimization of the accessible components of guideline-directed medical therapy (GDMT), specifically renin-angiotensin system inhibitors (RASIs), beta-blockers, and mineralocorticoid receptor antagonists.

## Methods

### Aim

The objective of the study is to evaluate the implementation of guideline-directed medical therapy (GDMT) and identify associated clinical factors among patients with heart failure with reduced ejection fraction (HFrEF) receiving follow-up at Asella Referral and Teaching Hospital, Ethiopia.

### Study Period and Area

A cross-sectional study of 156 HFrEF patients (LVEF ≤ 40%, ≥ 6 monthly visits) from October–December 2023. Asella Referral and Teaching Hospital (ARTH) is a tertiary-level referral hospital affiliated with Arsi University, located in Asella town, approximately 175 km southeast of Addis Ababa. It serves a catchment population of over 3.5 million people in the Arsi Zone and surrounding regions, offering specialized inpatient and outpatient services, including chronic heart failure management.

The Internal Medicine Department offers both inpatient and outpatient care, with dedicated outpatient clinics for chronic disease management, including heart failure follow-up services. The Adult Medical Clinic operates on scheduled days and manages patients with heart failure, hypertension, arrhythmias, ischemic heart disease, and other cardiovascular conditions. Patients enrolled in this study were those attending the heart failure follow-up service, where regular clinical evaluations, echocardiographic assessments, and medication titrations were conducted in accordance with guideline-directed management strategies.

## Populations

### Source population

The source population consists of all heart failure patients visiting the referral clinic during the study period, while the study population includes HFrEF patients who met the inclusion criteria and gave their consent.

### Eligibility criteria

The study enrolled adult patients (> 18 years) with heart failure with reduced ejection fraction (HFrEF). HFrEF was defined as LVEF ≤ 40% (on echocardiography performed within the previous two years, who had completed at least six consecutive monthly follow-up visits at the cardiac clinic) plus documented symptoms (e.g., dyspnea) or signs (e.g., edema) at diagnosis. NYHA Class I status reflected clinical stability at follow-up, not absence of prior symptoms. Exclusion criteria consisted of: [1] unavailable or inconclusive echocardiographic data for HF classification [2], preserved LVEF (> 40%) [3], insufficient clinic attendance (< 6 visits), or [4] refusal to provide informed consent. From an initial screening pool of 175 potential participants, secondary review excluded 19 cases (15 due to inadequate follow-up duration and 4 lacking recent echocardiographic confirmation), resulting in a final study population of 156 patients who met al.l inclusion criteria.

### Sample size

The sample size was calculated by using a single population proportion formula using Epi-info version 7.2.

Z α/2 is a standard normal variant.

d = margin of error taken as 0.05.

p = an estimation of the prevalence of patients not prescribed beta blockers was 33% (*p* = 0.33) [12].

q = 1 - p: the probability of non-occurrence of the event of interest.

n = (1.96)(1.96)(0.33)(0.67) = 340.

(0.05)²

The calculated sample size was 340 patients.

n = corrected sample size.

n₀ = Initial sample size (340).

N = Source population size (175 HFrEF patients on follow-up).

Given the finite source population of 175 eligible HFrEF patients, we applied a population correction factor to our sample size calculation. The initial adjusted sample size of 116 increased to 128 after accounting for a 10% non-response rate. However, as this represented 73% of the eligible population (128/175), we opted for a census approach to maximize statistical power and eliminate sampling error. From the total population, we excluded 19 patients (15 with < 6 monthly visits and 4 lacking updated echocardiograms), yielding a final analytical sample of 156 patients (89% of the eligible population). This census method ensured complete representation of HFrEF cases at our institution while maintaining 98.7% of our target sample size. All included patients had complete primary outcome data (GDMT prescription patterns and dose achievement) for comprehensive analysis.

### Sampling procedure

All patients with heart failure with reduced ejection fraction (HFrEF) who were on regular follow-up at the Adult Medical Outpatient Clinic and who fulfilled the eligibility criteria were included in the study using a census method. Data collection took place during the study period.

### Variables

Dependent variable: GDMT implementation.

Independent variables: Sociodemographic factors: age, gender, marital status, education level, income, place of residence, and healthcare insurance usage; Clinical and laboratory-related characteristics: baseline HR and blood pressure, duration of diagnosis of HF, HF hospitalization, NYHA functional class, LVEF, underlying cause of heart failure, comorbidities, serum creatinine, and serum potassium; Health care and treatment-related factors: evidence-based/GDMT drugs’ dose, other prescribed medications, medication side effects, unavailability of drugs, and reason for not using GDMT at target dose.

### Operational Definition

Target doses based on AHA/ACC/HFSA 2022 and ESC 2021 guidelines [2, 3].

#### ARNI target dose

sacubitril/valsartan 93/103 mg bid (200 mg bid).

#### ACEIs target dose

lisinopril 20–40 mg/day, enalapril 10-20 mg bid, fosinopril 20 mg/day, captopril 50 mg tid, and ramipril 10 mg/day.

#### ARBs target dose

valsartan 160 mg bid, losartan 150 mg/day, and candesartan 32 mg/day.

#### Beta-blockers target dose

Metoprolol XR 200 mg/day, Carvedilol 25 mg bid or 50 mg bid for patients weighing > 85 kg, Bisoprolol 10 mg/day,

#### MRA target dose

Spironolactone 25–50 mg/day.

#### SGLT2Is

dapagliflozin 10 mg or empagliflozin 10 mg.

#### Ischemic heart disease

(IHD) was clinically defined by physician diagnosis supported by either: [1] ECG evidence of prior infarction [2], documented history of typical angina, or [3] prior records indicating ischemic etiology. Given limited angiography availability, alternative causes were excluded through clinical assessment.

### Data collection tools and procedures

Target doses for GDMT were defined per the 2022 AHA/ACC/HFSA and 2021 ESC Heart Failure Guidelines, which provide the most current evidence-based thresholds. While the Ethiopian STG [24] was consulted for local context, its recommendations were limited to older drug classes (e.g., ACE inhibitors) due to resource constraints. International guidelines were prioritized to benchmark care against global standards [2, 3].

A structured questionnaire was developed based on the 2022 AHA/ACC/HFSA guidelines [2, 3], and adapted for the Ethiopian context using findings from prior local studies [21, 24]. Sociodemographic variables (income, education, residence) were incorporated using the WHO STEPS instrument [25]. The English version underwent professional translation to Afan Oromo following ISPOR standards [26], with back-translation verification and pretesting conducted on 16 participants (10% of sample size) to ensure conceptual equivalence and cultural appropriateness before deployment. Socio-demographic information was gathered through face-to-face interviews using the questionnaire, while clinical, laboratory, and medication-related data were extracted from patient records. Data collection was carried out by nurses who received two days of training on the study procedures. The questionnaire is provided in Supplementary File 1. Missing clinical data were minimal (< 5%) and were excluded from multivariate analysis. Recall bias is a potential limitation, particularly for patient-reported variables.

### Data Quality Control

Prior to data collection, data collectors received a two-day orientation and training. The questionnaire was pretested on 10% of the sample size to ensure its effectiveness before the actual survey began. The principal investigator supervised the data collection process, and each questionnaire was reviewed daily for consistency, completeness, clarity, and accuracy.

### Data Processing and Analysis

The collected data were edited, cleaned, and organized appropriately. After coding, the data were entered into SPSS version 26 for analysis. Descriptive statistics, such as frequencies and proportions, were used to summarize the study population. Bivariate and multivariate logistic regression analyses were conducted to assess associations between dependent and independent variables. Variables with a p-value less than 0.25 in the bivariate analysis were included in the multivariable model to control for confounding. A p-value of less than 0.05 was considered statistically significant for identifying predictors of GDMT implementation.

## Results

Socio-demographic characteristics of the respondents.

Among 156 participants, 50.6% were female. The mean age of participants was 63.4 ± 6.5 years, and the mean estimated distance to the hospital was 37.95 ± 27.17 km. Most patients (68.6%) resided in rural areas, and 74.4% were married. Over half (53.2%) were farmers by occupation, and 89.7% of the participants had health insurance coverage (Table 1).

**Table 1:** Socio-demographic characteristics of patients with HFrEF attending the adult follow-up clinic at Asella Hospital, Southeast Ethiopia, 2023 (*n* = 156)

Variables	Category	Frequency (*n*)	Percentage (%)
Gender	Male	77	49.4
	Female	79	50.6
Marital status	Married	116	74.4
	Single	4	2.6
	Divorced	7	4.5
	Widowed	29	18.6
Educational level	No formal education	80	51.3
	Primary school	51	32.7
	High school	9	5.8
	Diploma	4	2.6
	Degree and above	12	7.7
Occupation	Student	2	1.3
	Government employee	20	12.8
	Merchant	13	8.3
	Housewife	38	24.3
	Farmer	83	53.2
Residence	Urban	49	31.4
	Rural	107	68.6
Monthly income in ETB	< 2000	94	60.3
	2,000–5,000	35	22.4
	5000-10,000	16	10.3
	> 10,000	11	7.1
Health insurance coverage	Yes	140	89.7
	No	16	10.3

### Clinical and laboratory-related characteristics of the study participants

Among 156 patients included in the study, 27 (17.3%) have hypertension at the time of the interview. The majority of the patients, 135 (86.5%), have a normal baseline heart rate (60–100). Ischemic heart disease was the most common underlying cause of heart failure (89.7%), while only 13 patients (8.3%) had dilated cardiomyopathy (DCM). The mean ± ± SD of duration since heart failure diagnosis was 21.83 ± 27.61 months, while the mean ± ± SD of guideline-directed medical therapy (GDMT) initiation was 20.49 ± 11.35 months. More than half of the patients, 82 (52.6%), started on guideline-directed medical therapy (GDMT) immediately after HF diagnosis, but 47.4% initiated it after 14 14months of HF diagnosis. On the other hand, the majority, 93 (59.6%), of patients have a history of previous hospitalization due to heart failure. Regarding the functional status of the participants, 94 (60.3%) were in NYHA class I functional state, while 51 (32.7%) were in class II. Left ventricular systolic function was moderately reduced (ejection fraction 31–40%) among 80 (51.3%) of patients, while 76 (48.7%) had severely reduced left ventricular systolic function (EF = 30% or below). Regarding the serum creatinine, 14 (9%) had elevated serum creatinine levels, while the remaining had normal serum creatinine levels. Most study participants, 128 (82.1%), had normal serum potassium levels, while hyperkalemia and hypokalemia were identified in 18 (11.5%) and 10 (6.4%) of cases, respectively (Table 2).

**Table 2:** Clinical and laboratory parameters of patients with HFrEF attending the adult follow-up clinic at Asella Hospital, Southeast Ethiopia, 2023 (*n* = 156)

Variable	Category	Frequency	Percentage
Blood pressure	Hypertensive	27	17.3
	Hypotensive	10	6.4
	Normotensive	119	76.3
Baseline heart rate	Bradycardic	9	5.8
	Normal HR	135	86.5
	Tachycardic	12	7.7
Underlying cause of HF	IHD	140	89.7
	DCM	13	8.3
	HHD	3	1.9
Duration of HF diagnosis (months)	6–23	44	28.2
	24–40	58	37.2
	41–57	19	12.2
	58–74	20	12.8
	75–144	15	9.6
Duration of GDMT initiation (month)	6–12	52	33.3
	13–24	55	35.2
	25–36	37	23.7
	37–60	12	7.8
Delay of GDMT initiation	Without delay	82	52.6
	1-6months	12	7.7
	7–12 months	17	10.9
	> 12 months	45	28.8
Previous hospitalization for heart failure	Yes	93	59.6
	No	63	40.4
NYHA functional class of HF	class I	94	60.3
	class II	51	32.7
	class III	9	5.8
	class IV	2	1.3
Left ventricular ejection fraction	31–40%	80	51.3
	30% or less	76	48.7
Comorbid illnesses*	Yes	106	68
	None	50	32
Serum creatinine	Normal	142	91.0
	High(> 1.2 g/dl)	14	9.0
Serum potassium	Hyperkalemia	18	11.5
	Hypokalemia	10	6.4
	Normal	128	82.1

Note: Underlying causes of heart failure: IHD (ischemic heart disease), DCM (dilated cardiomyopathy), and HHD (hypertensive heart disease)

Other comorbid conditions assessed separately included hypertension, diabetes mellitus, atrial fibrillation, and others. GDMT: guideline-directed medical therapy.

### Medication prescription patterns

Prescription rates for ACE inhibitors, beta-blockers, and MRAs were 98.1%, 96.8%, and 70.5%, respectively. None of the participants received angiotensin receptor–neprilysin inhibitors (ARNI) or sodium-glucose co-transporter 2 inhibitors (SGLT2i). Enalapril and Losartan were the two RAAS inhibitors prescribed for patients. For 53 (98.1%) of the participants, RAAS inhibitors were prescribed. However, only 48 (30.8%) are taking the target dose of RAAS inhibitor (Enalapril 10 mg bid); the rest, 105 (67.3%), were taking the medication but not at guideline-recommended target doses. On the other hand, among the study participants, 151 (96.8%) were taking cardioprotective beta-blockers. Metoprolol extended release was the most commonly used medication, followed by carvedilol and bisoprolol. Among those, only 2 (1.3%) patients are taking cardio-selective beta blockers at target doses (bisoprolol 10 mg daily). Mineralocorticoid receptor antagonist (spironolactone) was prescribed for 110 (70.5%) of the participants; among those, the target dose (spironolactone 25–50 mg/day) was prescribed for 70 (67.3%). On the other hand, SGLT2I (dapagliflozin or empagliflozin) was not given to any of the participants (Table 3).

**Table 3:** Prescription rates and target dose achievement of GDMT components in patients with HFrEF attending adult follow-up clinic at Asella Hospital, Southeast Ethiopia, 2023 (*n* = 156)

Group of medications	Category	Frequency	Percentage
ACEI/ARB	Enalapril 2.5 mg po bid/losartan 25 mg po daily	13	8.3
	Enalapril 5-7.5 mg po bid/losartan 50-100 mg daily**	92	59.0
	Enalapril 10 mg po bid/losartan 150 mg/day**	48	30.8
	Not taking	3	1.9
BBs	Metoprolol XL 12.5-25 mg/day/carv. 3.125 mg bid/bisoprolol 2.5 mg daily	69	44.2
	Metoprolol XL 37.5-50 mg/carv. 6.25 mg bid/bisoprolol 5 mg daily	62	39.7
	Metoprolol XL 100/carv. 12.5 mg bid/bisoprolol 7.5 mg daily	18	11.5
	Bisoprolol 10 mg daily**	2	1.3
	Not taking	5	3.2
MRAs	Spironolactone 12.5 mg daily	5	3.2
	Spironolactone 25 mg daily**	103	66.0
	Spironolactone 50 mg daily**	2	1.3
	not taking Spironolactone	46	29.5
SGLT2Is	Dapagliflozin/Empagliflozin 10 mg po daily	0	0
	Not taking	156	100

Note: The asterisks (**) show the target dose for each medication

The reasons for the underutilization of different classes of medications were also assessed. The major reasons for not using SGLT2Is at the target doses were unaffordability, which accounts for 93.6%, followed by unavailability (6.4%). Even though the vast majority of patients, 151 (96.8%), in this study were prescribed BBs, only 2 patients (1.3%) were on the optimal dose. Some of the reasons were that when there was a hospital admission for heart failure, the prescribed BB was discontinued, and patients were restarted with lower doses, which required frequent up-titration to reach the guideline-directed target dose. Medication side effects and contraindications were also the reasons for 41 (12.1%) and 30 (8.8%) of the participants, respectively.

### Factors associated with guideline-directed medication therapy utilization among patients with HFrEF

Logistic regression analysis revealed factors significantly associated with target dose utilization of guideline-directed medication therapy. Among clinical, laboratory, and medication-related factors, baseline heart rate (bradycardia: AOR 13.7 (1.8-100.3), P-value 0.010) and having diabetes mellitus (AOR 4.5 (1.13–17.9), P-0.033) as a comorbidity are significantly associated with the use of RASI at the target dose (Table 4).

**Table 4:** RASI target dose and associated factors among patients with HFrEF attending the adult follow-up clinic at Asella Hospital, Southeast Ethiopia, 2023 (*n* = 156)

Variable	Category	RASI at target dose		COR(95% CI)	P-value	AOR(95% CI)	P-value
		Yes	No				
Blood pressure	Normotensive	33	86	1		1	
	Hypotensive	1	9	0.29(0.35-2.37)	0.248	0.18(0.002-16.4)	0.46
	Hypertensive	14	13	2.81(1.19–6.6)	0.018	3.3(0.902 − 12.1)	0.071
Baseline Heart rate	Normal	39	96	1		1	
	Bradycardia	7	2	8.6(1.71–43.3)	0.009	13.7(1.8-100.3)	0.010*
	Tachycardia	2	10	0.49(0.1-2.35)	0.374	0.71(0.113 − 4.4)	0.709
Comorbidity	Hypertension	17	28	1.56(0.75-3.26)	0.229	0.52(0.162 − 1.67)	0.273
	Diabetes	10	8	3.3(1.21–8.96)	0.02	4.5(1.13–17.9)	0.033*
	Atrial fibrillation	4	22	0.35(0.12 − 1.1)	0.072	0.38(0.095 − 1.5)	0.168
Drug SE	hypotension	1	10	0.21(0.026-1.67)	0.141	0.85(0.01-66.7)	0.944
	No	20	30	1	1	1	

*statistically significant

### Factors associated with the use of mineralocorticoid receptor antagonists

Our analysis identified both class-specific and cross-cutting predictors of GDMT optimization. Three overarching themes emerged: [1] prior hospitalization predicted target-dose achievement for both MRAs (AOR = 0.4; 95% CI: 0.18–0.9; *p* = 0.029) and RASIs [2], renal dysfunction consistently limited up-titration across all drug classes, and [3] provider specialty influenced dosing decisions. For MRAs specifically, hypertension comorbidity was a significant predictor, with non-hypertensive patients having 63% lower odds of receiving target doses (AOR = 0.37; 95% CI: 0.14–0.99; *p* = 0.050) (Table 5). These findings suggest that while clinical factors like hospitalization history and comorbidities drive medication-specific decisions, system-level barriers (e.g., renal monitoring capacity) uniformly constrain optimal GDMT implementation.

**Table 5:** Factors associated with GDMT optimization (including MRA target dosing) among patients with HFrEF attending adult follow-up clinic at Asella Hospital, Southeast Ethiopia, 2023 (*n* = 156)

Variable	Category	MRA at target dose		COR(95% CI)	AOR(95% CI)	P-value
		Yes	No			
Level of education	No formal educ.	56	24	1	1	
	Primary school	30	21	0.61(0.29-1.28)	0.61(0.26-1.39)	0.239
	High school	8	1	3.4(0.41-28.9)	7.5(0.74-76.4)	0.088
	Diploma	1	3	0.14(0.14-1.44)	0.16(0.013 − 1.9)	0.147
	Degree and above	10	2	2.14(0.436 − 10.5)	3.75(0.64-21.8)	0.142
Blood pressure	Normotensive	86	33	1	1	
	Hypotensive	4	6	0.25(0.068-0.965)	0.23(0.015 − 3.3)	0.279
	Hypertensive	15	12	0.48(0.2-1.13)	0.84(0.27 − 2.6)	0.766
Previous HF hospitalization	Yes	67	26	0.59(0.299 − 1.16)	0.4(0.18–0.9)	
	No	38	25	1	1	0.029*
Comorbidity	Hypertension (yes)	24	21	0.24((0.10–0.58)	0.37(0.14–0.99)	
	Hypertension (No)	12	43	1	1	0.050

*statistically significant

## Discussion

Heart failure with reduced ejection fraction (HFrEF) remains a major global health challenge, characterized by high morbidity and mortality [4]. This study assessed the implementation of guideline-directed medical therapy (GDMT) among patients with heart failure with reduced ejection fraction (HFrEF) at Asella Referral and Teaching Hospital. Adherence to optimal GDMT, particularly achieving target medication doses, was found to be substantially low.

At the time of data collection, angiotensin receptor-neprilysin inhibitors (ARNIs) and sodium-glucose co-transporter-2 inhibitors (SGLT2i) were neither available in Ethiopian government supply chains nor affordable through private pharmacies. These agents remain excluded from the Ethiopian Standard Treatment Guidelines [24], explaining their absence in this cohort.

This study involved a mean age of 63.4 ± 12.56 years, which is younger compared to global data (72.3 ± 11.8 years) [16] and almost similar to a study done in Oman [8] in which the mean age was 63 ± 15 years. This is probably due to the lower Life expectancy in low- and middle-income countries compared to data from the high-income countries. The majority of patients are from rural areas of the country and have no formal education, which is also in Line with most of the local data, including the 2019 mini demographic and health survey (DHS) report [7, 9, 10, 17].

The most common underlying cause of heart failure in this study was ischemic heart disease (89.7%). A study done at Tikur Anbesa Hospital also shows that IHD is the most common underlying cause of heart failure, followed by cardiomyopathy [7]. Almost half of the patients have a left ventricular ejection fraction that is 30% or less. Those patients with severe left ventricular systolic dysfunction (LVEF ≤ 30) were hospitalized due to heart failure more than once in the past year. Although prescription rates for RAS inhibitors, beta-blockers, and mineralocorticoid receptor antagonists (MRAs) were relatively high, achieving target doses remained Limited. Only 30.8% of patients achieved the target dose of RASIs, 1.3% reached target-dose beta-blockers, and 67.3% achieved target-dose MRAs. Notably, no patients in this cohort received ARNIs or SGLT2 inhibitors. At the time of data collection, these medications were neither included in the Ethiopian Standard Treatment Guidelines (STG) [24] nor available in public supply chains. Private procurement was prohibitively expensive, with 93.6% of participants citing unaffordability as the primary barrier. This highlights a critical gap between international GDMT recommendations and local formulary realities in resource-limited settings. These findings are consistent with prior reports from resource-limited settings, where access barriers and healthcare system constraints limit full implementation of contemporary heart failure guidelines [10, 11].

Our study found 70.5% (110/156) of HFrEF patients received MRAs, with 66% (68/103) of NYHA Class I patients prescribed these medications—a pattern diverging from international guidelines recommending MRAs primarily for NYHA II–IV. This discrepancy likely reflects three contextual factors: [1] clinical stabilization of previously symptomatic patients (59.6% had prior hospitalizations) [2], high-risk features in Class I patients (48.7% with LVEF ≤ 30%), and [3] local guideline flexibility, as the Ethiopian STG permits MRA use based on EF severity rather than symptom class. While this approach may represent rational adaptation to resource constraints, it underscores the need for standardized protocols balancing global evidence with local realities.

Baseline bradycardia was independently associated with achieving target doses of RASI, with an Adjusted Odds Ratio (AOR) of 13.7 (95% CI: 1.8–100.3, *p* = 0.010). This finding likely reflects that RAS inhibitors, unlike beta-blockers, do not lower heart rate, and therefore their titration is less limited by pre-existing bradycardia. Patients with lower resting heart rates may tolerate RASI up-titration better, without concerns of symptomatic hypotension induced by additional bradycardia. This aligns with observations from prior pharmacological studies of GDMT implementation [16–18]. Clinicians might also prioritize RASIs over other heart failure medications in such cases due to their well-established mortality and morbidity benefits, especially in the absence of contraindications. This careful therapeutic selection and monitoring may explain the higher likelihood of RASI prescription in patients with baseline bradycardia.

Diabetes mellitus was also significantly associated with achieving the target dose of RASI (AOR 4.5, 95% CI: 1.13–17.9, *p* = 0.033). This finding is consistent with findings from both global and regional studies. RASIs are well-recognized for their dual benefits in patients with heart failure and diabetes, offering not only symptomatic relief and improved cardiac remodeling but also nephroprotection. This finding aligns with studies conducted in high-income countries, such as those by Komajda et al. (2011) [19] and Yancy et al. (2017) [20], where diabetic patients were significantly more likely to receive RASI due to their elevated cardiovascular risk and the protective benefits of RASI. Similarly, research from sub-Saharan Africa and Ethiopia (Adamseged et al., 2024) [21] has shown increased RASI use among diabetic heart failure patients, reflecting clinician adherence to global evidence-based practices, even in Low- and middle-income countries. The renoprotective effects of RASI, particularly in diabetic patients, could explain this association. Diabetic patients are often at higher risk of developing kidney dysfunction, which is a common comorbidity in HFrEF, and RASI therapy plays a vital role in mitigating these risks. Thus, clinicians may be more inclined to achieve target doses in diabetic patients to maximize both cardiovascular and renal benefits.

Conversely, the absence of a history of heart failure hospitalization was associated with 60% lower odds of achieving the target MRA dose (AOR = 0.4, 95% CI: 0.18–0.9; *p* = 0.029), highlighting a critical gap in the optimization of guideline-directed medical therapy (GDMT). Patients who have been recently hospitalized may receive closer follow-up and more intensive medication adjustments. Additionally, MRAs offer not only mortality and morbidity benefits in HFrEF but also provide mild diuretic effects and potassium-sparing properties. These pharmacologic benefits make MRAs particularly attractive in patients recovering from acute decompensations, where fluid management and prevention of hypokalemia are important clinical goals. This likely contributed to the higher rates of MRA optimization observed among patients with a history of hospitalization. This pattern is consistent with several prior studies. For instance, the CHAMP-HF registry in the U.S. reported that patients recently discharged from hospitalization were more likely to receive appropriate GDMT, including MRAs, compared to stable outpatients [22]. Similarly, a study from Europe (ESC-HF Long-Term Registry) found that prior hospitalization was independently associated with higher rates of optimized MRA therapy, likely due to closer follow-up and more aggressive management strategies during and after hospitalization [23]. Furthermore, patients without hypertension were 63% less likely to receive the target MRA dose compared to those with hypertension (AOR = 0.37, 95% CI: 0.14–0.99, *p* = 0.050). This finding aligns with several previous studies that have reported a higher likelihood of MRA prescription in hypertensive heart failure patients. A study conducted by Komajda et al. (2011) in the ESC-HF Pilot Survey found that hypertension was positively associated with MRA use [19]. Similarly, the CHAMP-HF registry in the United States reported that patients with comorbid hypertension were more likely to be prescribed MRAs at target doses [6].

Most patients (60.3%) were NYHA Class I at follow-up, yet only 1.3% achieved target GDMT doses. This suggests clinical stability may inadvertently reduce dosing intensity, despite guideline recommendations for GDMT irrespective of symptom class. Beyond medication cost and availability, physician-level barriers such as hesitancy in up-titration due to concerns about side effects, lack of frequent follow-up appointments, and reliance on outdated practices may limit GDMT optimization. Additionally, systemic issues: such as lack of decision-support tools or heart failure clinics contribute to suboptimal implementation.

Our findings must be interpreted within the constraints of Ethiopia’s healthcare system, where structural barriers significantly influenced GDMT implementation. Despite high prescription rates for foundational therapies, only 1.3% of patients achieved target doses, and advanced agents like ARNI/SGLT2i were entirely absent; findings inextricably linked to three systemic challenges: [1] medication affordability (93.6% cited prohibitive SGLT2i costs) [2], inconsistent availability of target-dose formulations due to supply chain limitations, and [3] workforce constraints where general internists, not cardiologists, delivered most HF care. Even among insured patients (89.7%), coverage gaps for non-formulary drugs persisted. These realities highlight a critical implementation paradox: while clinicians demonstrated awareness of GDMT principles through high prescription rates, systemic barriers prevented optimal execution. This underscores the need for policy-level interventions addressing drug accessibility, provider training, and insurance reform to bridge guidelines-to-practice gaps in resource-limited settings.

## Conclusion

The implementation of guideline-directed medical therapy in HFrEF patients was suboptimal, with only 1.3% achieving target doses of all three available therapies. No patients received ARNI or SGLT2 inhibitors due to availability and cost barriers. Key clinical factors such as baseline heart rate, diabetes, and hospitalization history influenced medication optimization. GDMT optimization in HFrEF remains inadequate in Ethiopia. Policy interventions (e.g., formulary expansion) and structured titration protocols are urgently needed.

### Recommendations

Clinical Practice:

Introduce nurse-led or pharmacist-supported medication titration clinics.

Regular training of internists and general practitioners in updated GDMT protocols.

Policy and Health System:

Advocate for inclusion of ARNI and SGLT2i in the Ethiopian STG and national Essential Medicines List.

Strengthen procurement and distribution systems to reduce drug stock-outs.

Research:

Conduct multi-center, prospective studies with a larger population and longer follow-up. Include health system, economic, and provider-level factors in future analyses.

### Limitations

This study was conducted at a single tertiary hospital, which may limit the generalizability of the findings to other settings, particularly rural or primary care facilities. The cross-sectional design prevents us from assessing long-term outcomes or changes in therapy over time. Some associations had wide confidence intervals due to the modest sample size, which may affect the statistical power of subgroup analyses. Additionally, key diagnostic biomarkers such as NT-proBNP were unavailable, and some data obtained through patient interviews may be subject to recall bias. This study did not assess HF with improved EF (HFimpEF), which may have excluded patients who had EF recovery beyond 40% prior to data collection. IHD diagnoses relied on clinical criteria rather than coronary angiography, which may have introduced misclassification bias given the known challenges of differentiating ischemic from non-ischemic cardiomyopathy in resource-limited settings. Despite these limitations, the study provides meaningful insights into GDMT use in a low-resource context.

## Supplementary Information

Supplementary Material 1

## Data Availability

The datasets used and/or analyzed during the current study are available from the corresponding author on reasonable request.

## Abbreviations

ACC: American College of Cardiology
ACEIs: angiotensin—converting enzyme inhibitors
A.FIB: Atrial Fibrillation
AHA: American Heart Association
ARBs: angiotensin II receptor blockers
ARNI: angiotensin receptor neprilysin inhibitor
BBs: beta blockers
DCM: Dilated Cardiomyopathy
DM: Diabetes Mellitus
ESC: European Society of Cardiology
Ethiopian STD: Ethiopian Standard Treatment Guideline
HFimpEF: Heart failure with improved ejection fraction
HICs: High—Income Countries
HFpEF: Heart failure with preserved ejection fraction
HFrEF: Heart failure with reduced ejection fraction
LMICs: Low—and Middle—Income Countries
LVEF: Left ventricular ejection fraction
MRA: Mineralocorticoid receptor antagonists
NYHA: New York Heart Association
RASI: renin angiotensin aldosterone inhibitors
TTE: trans—thoracic echocardiography
VHD: Valvular Heart Disease
