# Supplementary material for: Implementation of Guideline-Directed medical therapy and factors in heart failure with reduced ejection fraction
Source: BMC Cardiovasc Disord. 2025 Oct 9;25:728. doi: 10.1186/s12872-025-05187-5 (PMC12512486; doi:10.1186/s12872-025-05187-5)
Supplement: Supplementary file 1 — Supplementary Material 1 [file 12872_2025_5187_MOESM1_ESM.docx]

**Part I Sociodemographic data**

| No | Variables | Response/category |
| --- | --- | --- |
| 101 | Card number(MRN) | ________________ |
| 102 | Age(yrs) | _______________ |
| 103 | Gender | 1. Male  2. Female |
| 104 | Marital status | 1. Married 3. Divorced  2. Single 4. Widowed |
| 105 | Educational level | 1. No formal education  2. Primary school  3. High school  4. Diploma  5. Degree and above |
| 106 | Occupation | 1. Student  2. Government employee  3. Merchant  4. Housewife  5. Farmer  6. Other:_____________ |
| 107 | Monthly income (ETB) | 1. <2,000  2. 2,000-5,000  3. 5,000-10,000  4. >10,000 |
| 108 | Place of residence | 1. Urban  2. Rural |
| 109 | Estimated distance from hospital(KM) | ____________________ |
| 110 | Usage of healthcare insurance | 1. Yes  2. No |

**Part II: Clinical and laboratory related characteristics**

| No | Variables | Response |
| --- | --- | --- |
| 201 | Blood Pressure(mmhg) | ______________ |
| 202 | Baseline Heart rate(bpm) | ______ |
| 203 | Underlying cause of heart failure | 1. IHD  2. DCMP  3. HHD  4. others(specify)________ |
| 204 | Duration since HF diagnosis(in years/months) | _________ |
| 205 | How long since the patient was started on GDMT(in years/months) | _______________ |
| 206 | Previous hospitalization due to heart failure: | 1. Yes  2. No |
| 207 | If your answer is yes, how many times in the past 1 year? | _____ |
| 208 | NYHA functional class at the time of interviewing | 1. Class I 3. class III  2. Class II 4. class IV |
| 209 | LVEF | 1. 31-40%  2. 30% or less |
| 210 | Comorbidities | 1. HTN 7. Anemia  2. Asthma/COPD 8. Hyperthyroidism  3. VHD 9. PAD  4. DM 10. Dyslipidemia  5. A.Fib 11. stroke  6. CKD 12. others(specify)________  13. None |
| 211 | Serum Creatinine (mg/dl) | _______ |
| 212 | Serum potassium(mEq/L) | ___________ |

**Part III: medication related factors**

| No | Variables | Response |
| --- | --- | --- |
| 301 | Use of GDMT drugs and their dose | 1. ARNI/ACEI/ARB____________________________  2. BB________________________________  3. Spironolactone_________________________  4. Dapagliflozin/Empagliflozin____________ |
| 302 | Other prescribed medications | 1. Loop diuretics 4. Digoxin  2. Aspirin 5. Warfarin  3. Statin 6. Others(specify)_____________ |
| 303 | Medication-related side effects | 1. Hypotension 4. AKI  2. Hyperkalemia 5. Dry cough  3. Bradycardia 6. Others(specify)______________  7. None |
| 304 | Reason for not up-titrating GDMT drugs to a target dose | 1. frail elderly  2. advanced heart failure(NYHA class IV)  3. Medication side effect  4. Contraindications to drug use  5. unavailability of the drugs  6. cannot afford  7. others(specify)____________________  8. not identified |
